# Multidimensional Quality Assessment of Guizhou Teas Integrating Thermogravimetric, Amino Acid, and Trace Element Analyses

**DOI:** 10.1002/fsn3.72431

**Published:** 2026-09-30

**Authors:** Libing Zhou, Chunli Huang

**Affiliations:** ^1^ Guangxi Science & Technology Normal University Laibin Guangxi China

**Keywords:** amino acid, ICP–OES, OPLS–DA, tea quality assessment, thermogravimetric analysis

## Abstract

The constituents of tea leaves are crucial determinants of the quality and nutritional value of tea. Traditional evaluation methods, which depend on sensory assessment or isolated indicators, have certain limitations. Consequently, this project aimed to develop a multidimensional and systematic comprehensive evaluation system for assessing tea nutrition and quality. In this study, the quality of five tea varieties from Guizhou, China, namely, Qingyu tiny kuding tea, Guiding yunwu tribute tea, Suiyang mountain silver flower tea, Shiqian moss tea, and Pu'an black tea, was comprehensively evaluated by integrating combustion heat, combustibility/combustion stability, ash, crude fiber, fat, trace element (ICP–OES), amino acid (A300 analyzer), and thermogravimetric parameter information. The results indicated that Qingyu tiny kuding tea was the highest quality, followed by Shiqian moss tea, according to the results of the multi‐indicator evaluation (*F* = −0.5711–0.9953). Gray pattern recognition (GPR) revealed that the most stable combustion occurred for Suiyang Mountain silver flower tea, followed by Guiding Yunwu tribute tea, Shiqian moss tea, Qingyu tiny kuding tea, and Pu'an black tea (*F* = 0.8102–0.8023). EFCA revealed that the tea samples could be categorized into three distinct groups, while the 38 variables could be classified into three clusters. OPLS–DA achieved strong discrimination (*R* ^2^ *X* = 0.887) and identified 21 key differentiators (VIP > 1) as key differentiators among the five tea types. This study provides a solid scientific foundation for the large‐scale development of tea resources, research on tea classification, and the comprehensive evaluation of tea quality in Guizhou, China.

## Graphical abstract

Five tea varieties from Guizhou, China, were comprehensively evaluated using combustion heat/stability, ash, crude fiber, fat, trace elements (ICP‐OES), amino acids, and thermogravimetric parameters. Multi‐indicator evaluation ranked Qingyu tiny kuding tea highest (*F* = −0.5711–0.9953), followed by Shiqian moss tea, Suiyang mountain silver flower tea, Pu'an black tea, and Guiding yunwu tribute tea. Gray pattern recognition showed Suiyang mountain silver flower tea had the most stable combustion (*F* = 0.8023–0.8102). OPLS–DA achieved strong discrimination (*R* ^2^ *X* = 0.887) and identified 21 key differentiators (VIP > 1).

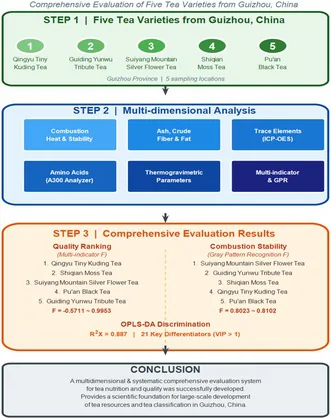

## 1 Introduction

The components of tea leaves are essential determinants of the quality and nutritional value of tea. Tea quality significantly influences consumer satisfaction and market demand. Consequently, the analysis and evaluation of tea quality are of paramount importance. Tea quality depends on various factors, including cultivation and processing techniques (Atila et al. 2026; Yang et al. 2026). Traditional evaluation methods, which rely on sensory assessments or isolated indicators, have inherent limitations. In addition, tea quality is influenced by various external factors, such as geographical location, climatic conditions, and harvest time (Cai et al. 2022; Huang et al. 2024; Lv et al. 2023). Guizhou, a significant tea‐producing region in China, is renowned for its diverse range of premium teas, which is largely attributed to its distinctive geographical and climatic conditions. Guizhou is situated at a high altitude with a temperate climate that is abundant in precipitation and characterized by fertile soil, which is conducive to tea tree growth (Deng et al. 2023; Jiang et al. 2023; Jin et al. 2018).

The presence of trace elements in tea positively contributes to the development of desirable characteristics, such as enhanced aroma and flavor. Conversely, elevated levels of heavy metals, including Pb and Cd, adversely affect tea quality and pose potential health risks to consumers. The amino acid composition of tea is crucial for determining the flavor profile and nutritional value of tea products. Furthermore, the concentration and balance of amino acids are influenced by tea variety, growth conditions, and processing methods. Consequently, amino acid analysis is an essential tool for tea producers and researchers aiming to optimize tea quality (Zhou and Huang 2025a, 2025b).

Thermogravimetric analysis (TGA) is extensively employed in the tea industry to investigate various aspects of tea quality and processing. TGAs offer valuable insights into the thermal decomposition behavior of tea leaves, thereby assisting researchers and manufacturers in understanding the transformations that occur during different stages of tea production. This method is particularly advantageous for analyzing the moisture content, volatile compounds, and overall thermal stability of tea samples, which are critical factors in determining the final quality and shelf life of tea products (Zhou, Jiang, Shi, and Jiang 2022).

Traditional sensory evaluation methods are characterized by subjectivity and require considerable time, with a notable deficiency in systematic evaluation studies concerning tea components (Zeng et al. 2023; Zhang et al. 2020). Consequently, these methods lack systematicity and comprehensiveness, hindering the scientific assessment of the diverse tea preferences of contemporary consumers. Addressing the challenge of conducting a comprehensive, multiparameter evaluation of tea quality and nutrition through objective and efficient chemometric methods has become a pressing issue in current tea research. In response to these challenges, this study presents innovative research on multiple indicators (Chen et al. 2022).

Thermal analysis elucidates the stability and decomposition properties of essential constituents, whereas amino acid profiling identifies primary contributors to flavor and potential bioactivity. Additionally, trace element analysis assesses both nutritional attributes and safety‐related factors. When integrated within a unified chemometric framework, these complementary datasets allow for more precise differentiation of teas that may appear similar under routine sensory or basic chemical evaluation (Zhou et al. 2026). This integration facilitates the identification of latent quality markers and provides a deeper understanding of the mechanisms underlying quality and grade differences. Consequently, this multidimensional strategy enhances the sensitivity, reliability, and explanatory power of tea quality evaluation beyond the capabilities of any single technique.

Compared with our previous studies, which focused on narrower analyte panels or more limited tea types, the present study offers a distinct and substantive advance in both scope and integration. By combining entropy factor analysis with OPLS–DA, we constructed a multidimensional quality and nutritional evaluation system that ranks teas more sensitively and objectively and enhances the interpretability of classification patterns in relation to cultivar traits and regional characteristics. This integrated multiparameter approach extends beyond our earlier publications by providing a more comprehensive and mechanistically informative basis for tea authentication, quality differentiation, and resource development in Guizhou (Lin et al. 2026).

It is imperative to evaluate tea quality using multiple indicators. By analyzing the presence of nutrients, such as fats, amino acids, and trace elements, in addition to potential health effects and other factors, a more comprehensive understanding of tea quality can be achieved. This multifaceted approach provides valuable insights into the overall quality and potential benefits of various types of tea (Chen et al. 2021; Ren et al. 2023; Song et al. 2021).

In this study, tea quality was comprehensively evaluated using chemometric analytical methods (Yu et al. 2023), with a focus on assessing the heat of combustion; the stability of combustion; and the contents of fat, crude fiber, ash, trace elements, and amino acids. Five types of tea leaves were examined in this study: Qingyu tiny kuding tea, Guiding yunwu tribute tea, Suiyang mountain silver flower tea, Shiqian moss tea, and Pu'an black tea. In this study, a multi‐indicator analysis and evaluation system for these tea leaves was developed, providing a solid scientific foundation for large‐scale studies of tea resource development and classification. This study aimed to provide theoretical and practical support for tea quality control and market positioning (Zhou and Zhang 2022).

## 2 Materials and Methods

### 2.1 Materials and Instruments

Five kinds of tea leaves—Qingyu tiny kuding tea, Guiding yunwu tribute tea, Suiyang mountain silver flower tea, Shiqian moss tea, and Pu'an black tea—were purchased from Guiyang Yunyan district farmers' markets in Guizhou, China, and were identified by Prof. Caiyun Jiang of Guangxi Science & Technology Normal University. Each sample underwent triplicate testing (*n* = 3, RSD% < 2%). All the samples were dried at 85°C for 12 h, finely ground using a mortar, sieved through a 40‐mesh pharmacopeia, and stored at Guangxi Normal University of Science and Technology.

Qingyu tiny kuding tea (origin: Yuqing County, Zunyi City, Guizhou Province, China; production date: May 2020), Guiding yunwu tribute tea (origin: Yunwu Town, Guiding County, Qiannan Buyi Miao Autonomous Prefecture, Guizhou Province, China; production date: September 2020), Suiyang mountain silver flower tea (origin: Suiyang County, Zunyi City, Guizhou Province, China; production date: September 2020), Shiqian moss tea (origin: Shiqian County, Tongren City, Guizhou Province, China; production date: March 2021), and Pu'an black tea (origin: Pu'an County, Buyei and Miao Autonomous Prefecture, Qianxinan, Guizhou Province, China; production date: March 2020).

A NETZSCH STA 2500 thermogravimetric analyzer (NETZSCH, Germany), an F1600 automatic fiber tester (Jinan Alva Instrument Co. Ltd.), an A300 amino acid analyzer (Germany Manmboer Company, Germany), and an ICP–OES spectrometer (iCAP 7000 SERIES, Thermo Scientific, USA) were used.

### 2.2 Determination Method for Each Index

The heat of combustion, ash content, fat content, trace element content, thermogravimetric parameters, and crude fiber content of the five teas were determined according to previous studies (Zhou and Hou 2024; Zhou, Jiang, and Lin 2022).

For the amino acid test method, 0.4 g of crushed and dried tea leaves were weighed, placed in a 50 mL volumetric flask, and mixed with 12 mmol/L hydrochloric acid to achieve a constant volume. After 10 min, the volumetric flask was shaken several times, a vacuum suction pump was used for suction filtration, and the filtrate was passed through a 0.22 μm membrane. At wavelengths of 570 and 440 nm, 20 amino acids were best separated under the separation conditions, the amino acid profiles were automatically generated, and the amino acid content was calculated by processing the peak areas at a later stage (Hagve et al. 2021; Liu et al. 2023). The chromatograms of the amino acid content in the tiny kuding tea of Qingyu are shown in Figures 1a and 1b. The contents of amino acids, such as CySO_3_H, Asp, MetSON, Thr, Ser, Glu, Gly, Ala, (Cys)_2_, Val, Met, Ile, Leu, Tyr, Phe, His, Lys, Thea, Arg, and Pro, were determined using an A300 amino acid analyzer (Zeng et al. 2015).

**FIGURE 1a:**
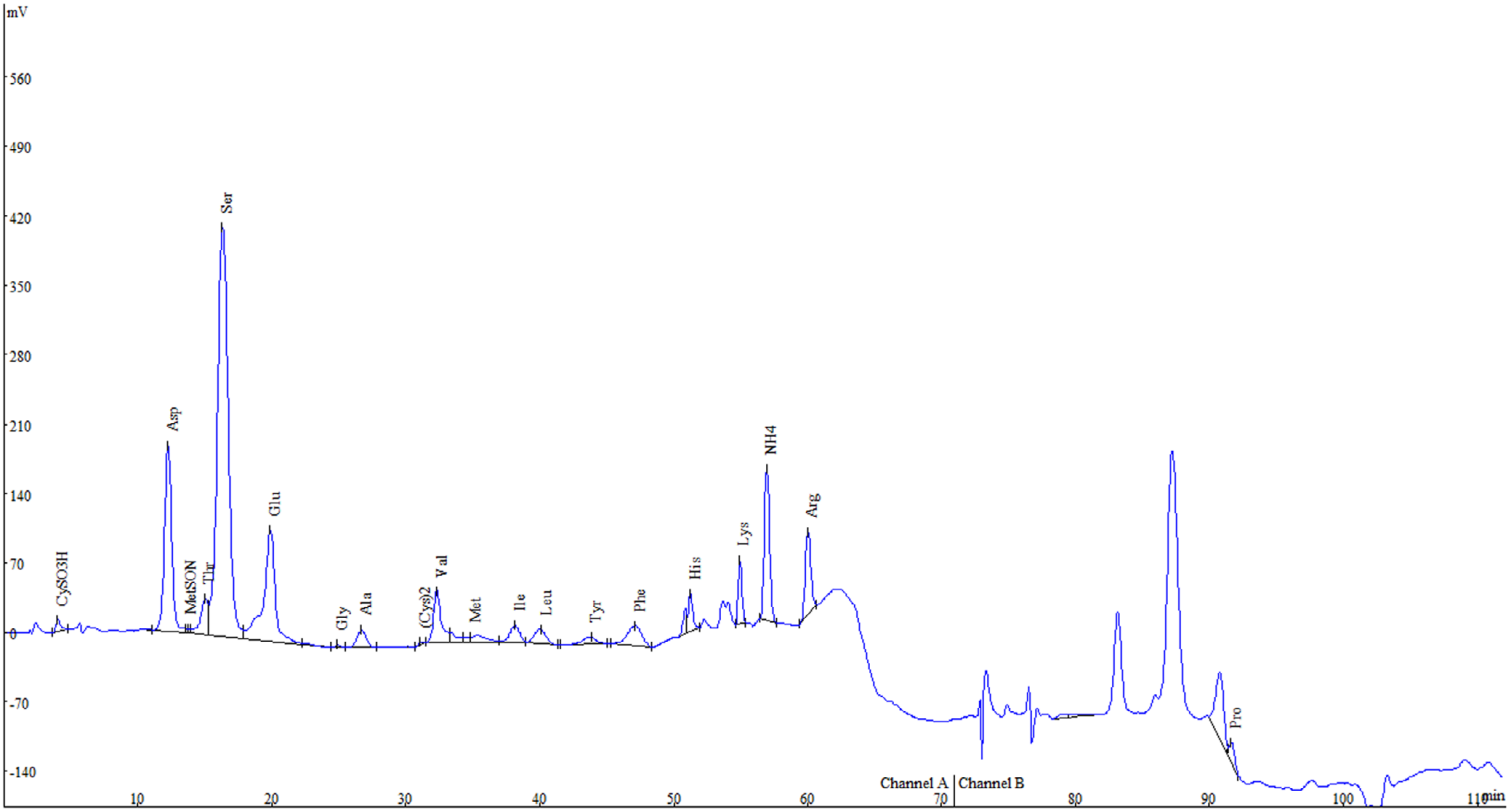
Chromatogram of the amino acid content of Qingyu tiny kuding tea.

**FIGURE 1b:**
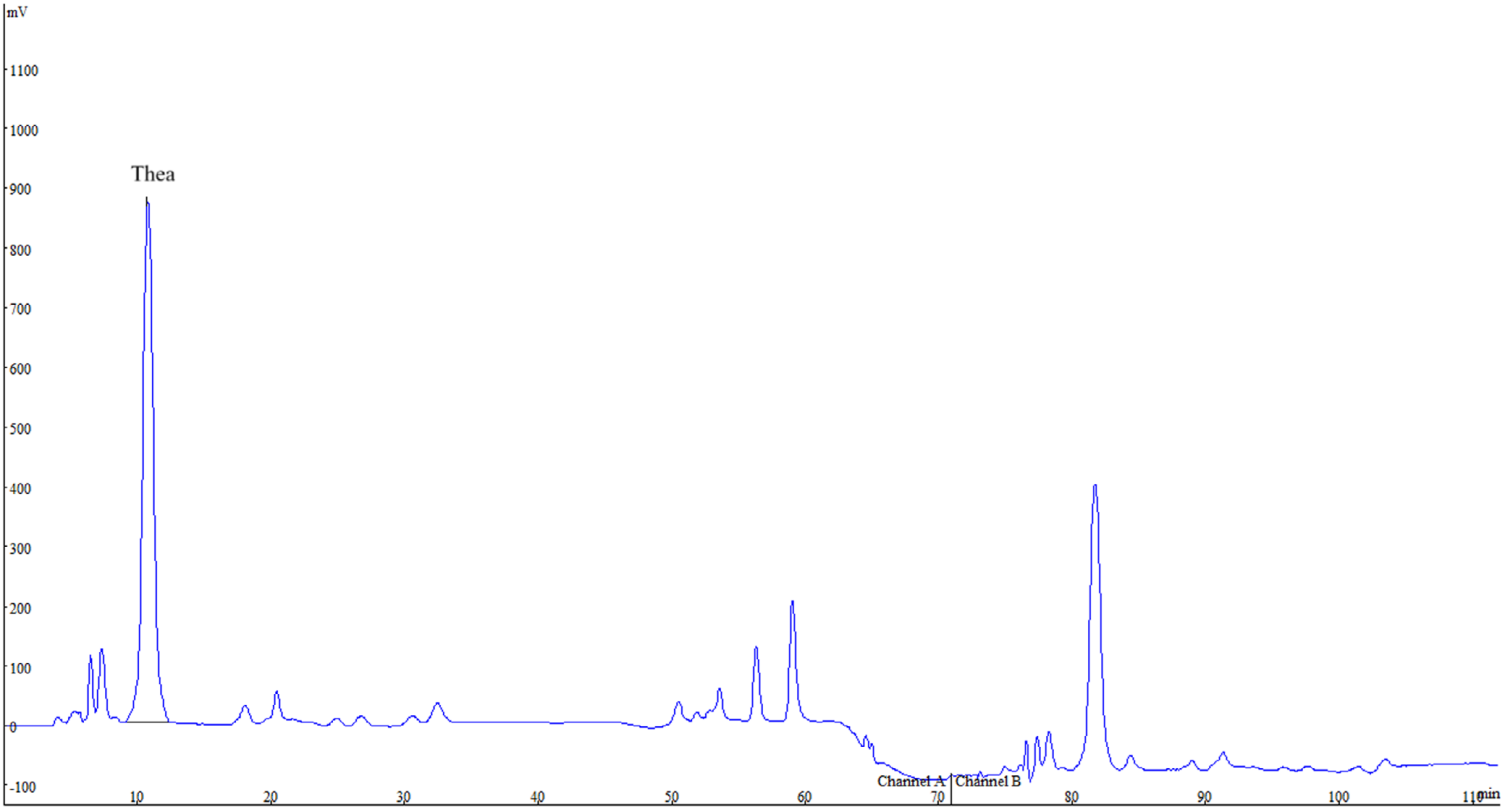
Chromatogram of the Thea content of Qingyu tiny kuding tea.

### 2.3 Multi‐Indicator Comprehensive Evaluation Method

On the basis of entropy factor analysis (EFA) and entropy factor cluster analysis (EFCA), comprehensive evaluation systems have been developed to analyze five types of tea leaves using multiple indicators, such as combustion heat, combustibility (stability of tea leaf combustion), fat content, ash, crude fiber, amino acids, and trace elements (Zhou and Huang 2025a, 2025b). The data were standardized to mitigate the effects of dimensionality and subsequently analyzed using the *p* test and leave‐one‐out cross‐validation (Austin et al. 2025).

## 3 Results and Discussion

### 3.1 Thermogravimetric Analysis

#### 3.1.1 Qingyu Tiny Kuding Tea

The thermogravimetric weight (TGW) of the tiny kuding tea from Qingyu is shown in Figure 2a, while the thermogravimetric analysis (TGA) data are presented in Table 1a. As shown in Figure 2a, the Qingyu tiny kuding tea sample began to decompose at 42.9°C, with a loss rate of 5.09% during the initial stage of decomposition. Upon reaching 133.6°C, the sample entered the second stage of decomposition, resulting in a loss rate of 56.68% at 430.1°C. The sample continued to decompose, and the final remaining mass was 28.11%.

**FIGURE 2a:**
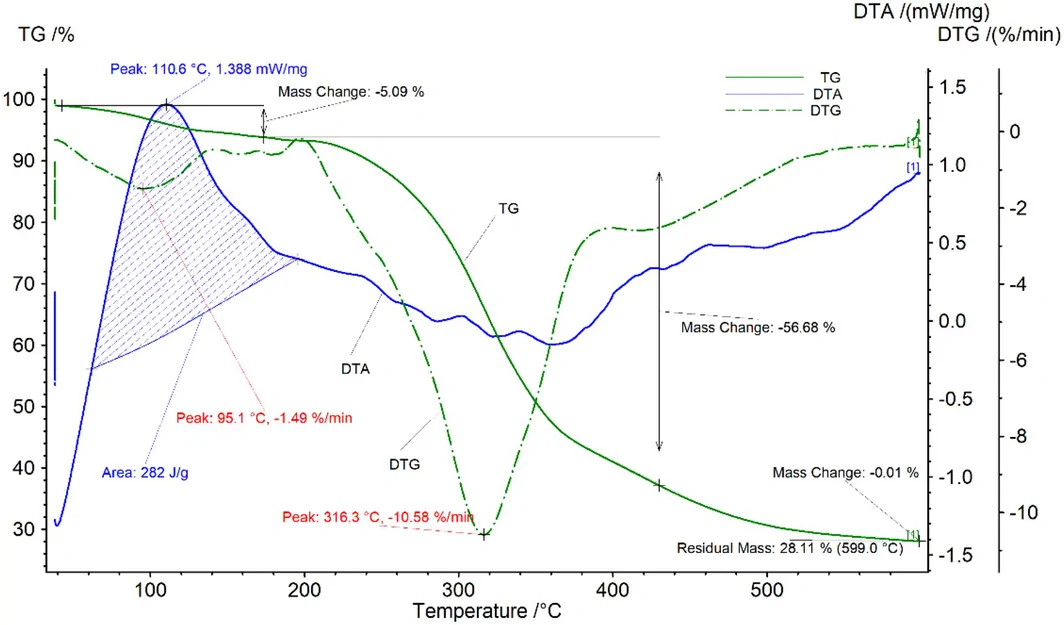
Thermogravimetric (TG) curve, differential thermal analysis (DTA) curve and differential thermal analysis (DTA) curve of Qingyu tiny kuding tea.

**TABLE 1a:** Thermogravimetric analysis data of the tiny kuding tea of Qingyu.

Curve	Project	Temperature range/°C	Percentage weight loss/%	Peak area/(J/g)	The fastest weight loss temperature/°C
TG, DTG	Peak 1	42.9~133.6	5.09	—	95.1
	Peak 2	133.6~430.1	56.68	—	316.3
DTA	Peak 1	61.9~195.7	—	282.00	110.6

The DTG curve of the Qingyu tiny kuding tea exhibited two peaks, with inflection points at 95.1°C and 316.3°C. The DTA curve of the Qingyu tiny kuding tea exhibited an exothermic peak at 110.6°C, with a temperature range from 61.9°C to 195.7°C and a peak area of 282.00 J/g.

#### 3.1.2 Guiding Yunwu Tribute Tea

The thermogravimetric weight of the Guiding yunwu tribute tea is shown in Figure 2b, and the thermogravimetric analysis data are presented in Table 1b. The temperature at which the Guiding yunwu tribute tea sample started to decompose was 60.3°C, and the loss rate was 4.43% after the first stage of decomposition (Figure 2b). When the temperature reached 174.5°C, the second stage of decomposition was initiated, and the loss rate was 63.20% at 424.4°C. The sample continued to decompose, and the final remaining mass was 18.39%.

**FIGURE 2b:**
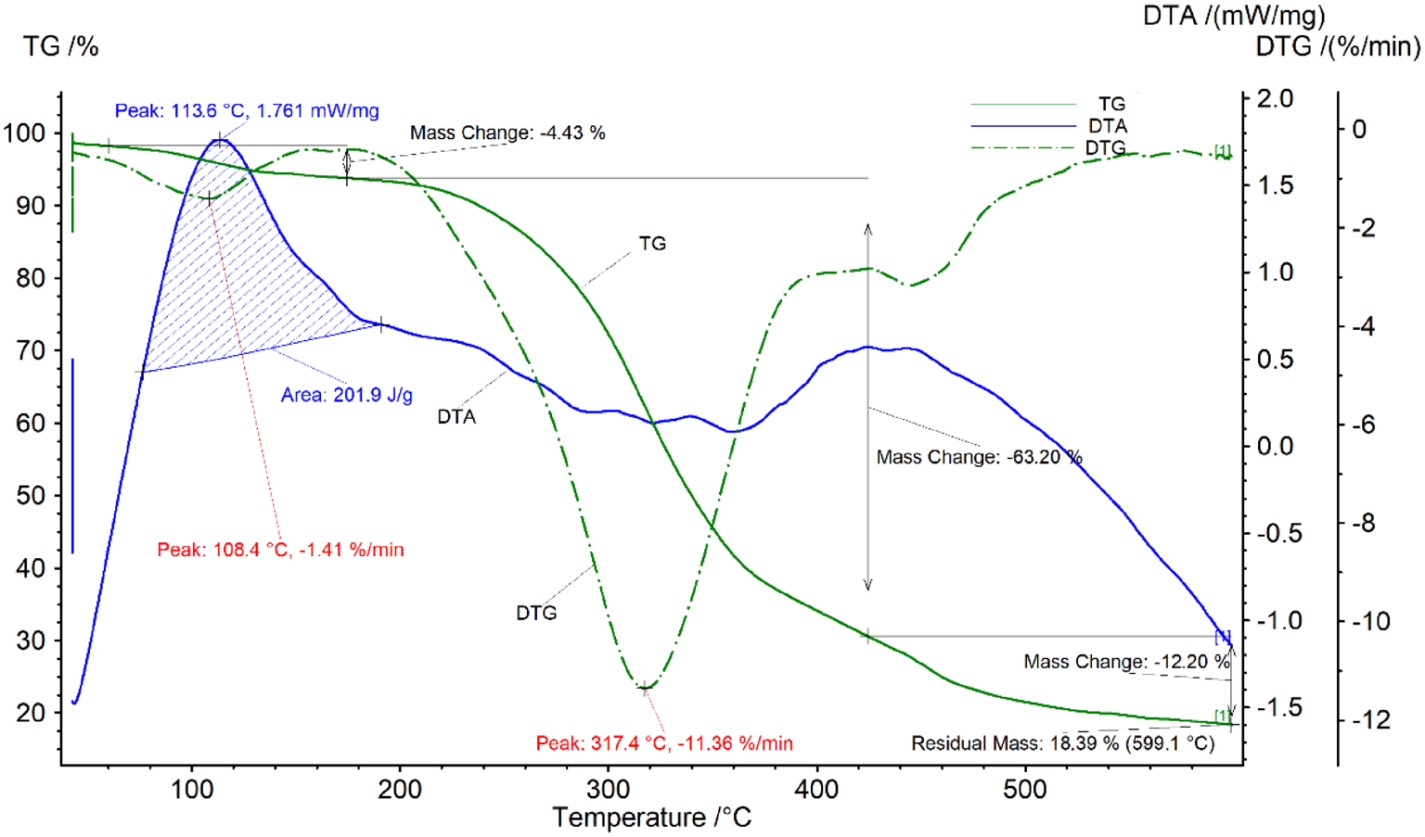
Thermogravimetric (TG) curve, thermogravimetric (DTG) curve and differential thermal analysis (DTA) curve of Guiding yunwu tribute tea.

**TABLE 1b:** TGA data of Guiding yunwu tribute tea.

Curve	Project	Temperature range/°C	Percentage weight loss/%	Peak area/(J/g)	The fastest weight loss temperature/°C
TG, DTG	Peak 1	60.3~174.5	4.43	—	108.4
	Peak 2	174.5~424.4	63.2	—	317.4
DTA	Peak 1	76.4~191.1	—	201.90	113.6

The DTG curve of Guiding yunwu tribute tea presented two peaks, with inflection points at 108.4°C and 317.4°C. The DTA curve of Guiding yunwu tribute tea exhibited a large exothermic peak, with a peak value of 113.6°C, a temperature range of 76.4°C–191.1°C, and a peak area of 201.90 J/g.

#### 3.1.3 Suiyang Mountain Silver Flower Tea

The results of the TG analysis of Suiyang mountain silver flower tea are shown in Figure 2c, and the corresponding data are listed in Table 1c. As shown in Figure 2c, the decomposition of the tea sample commenced at 51.6°C, with an 8.12% loss following heating in the initial stage. After reaching 167.5°C, the tea underwent a second stage of decomposition, which continued to reach 488.1°C, resulting in a mass loss of 49.35%. The remaining mass of the sample was 29.87%.

**FIGURE 2c:**
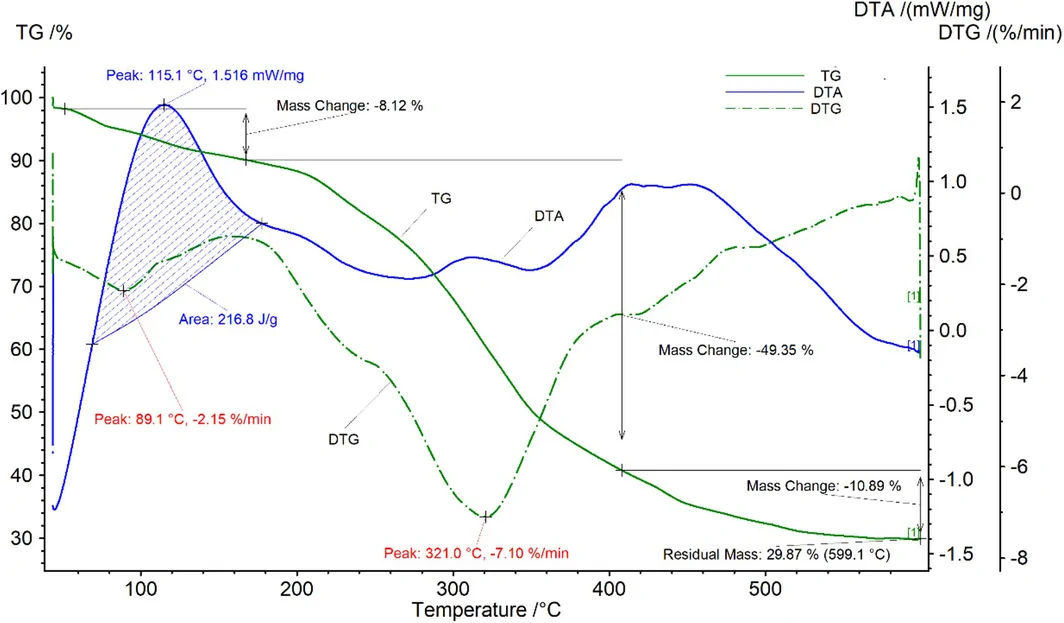
Thermogravimetric (TG) curve, thermogravimetric (DTG) curve and differential thermal analysis (DTA) curve of Suiyang mountain silver flower tea.

**TABLE 1c:** Thermogravimetric analysis data of Suiyang mountain silver flower tea.

Curve	Project	Temperature range/°C	Percentage weight loss/%	Peak area/(J/g)	The fastest weight loss temperature/°C
TG, DTG	Peak 1	51.6 ~ 167.5	8.12	—	89.1
	Peak 2	167.5 ~ 488.1	49.35	—	321.0
DTA	Peak 1	69.0 ~ 177.7	—	216.80	115.1

The DTG curve for Suiyang mountain silver flower tea exhibited two significant peaks at 89.1°C and 321.0°C. The DTA curve showed a prominent exothermic peak at 115.1°C, spanning a temperature range from 69.0°C to 177.7°C, with a peak area of 216.8 J/g.

#### 3.1.4 Shiqian Moss Tea

The TG data of Shichian moss tea are shown in Figure 2d, and the thermogravimetric analysis data are shown in Table 1d. The decomposition of the Shichian moss tea samples started at 43.3°C, and after the first stage of decomposition, the rate of loss was 5.02%, as shown in Figure 2d. When the temperature reached 172.2°C, it entered the second stage of decomposition, and the rate of loss was 44.89% when it reached 400.1°C. The samples continued to decompose, and the final residual mass was 36.83%.

**FIGURE 2d:**
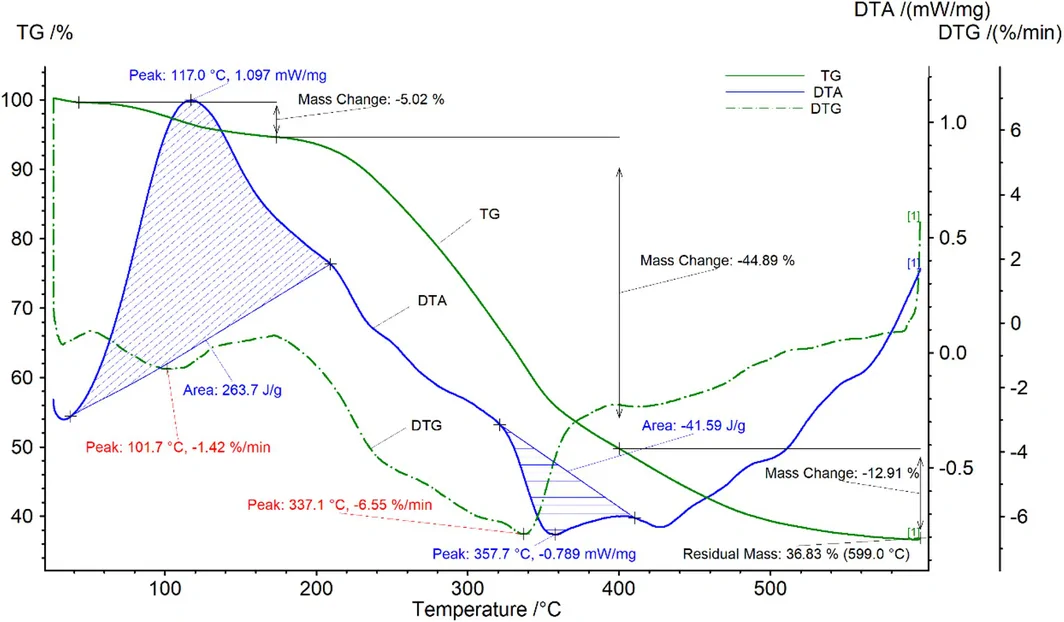
Thermogravimetric (TG), thermogravimetric (DTG), and differential thermal analysis (DTA) curves of Shiqian moss tea.

**TABLE 1d:** Thermogravimetric analysis data for Shiqian moss tea.

Curve	Project	Temperature range/°C	Percentage weight loss/%	Peak area/(J/g)	The fastest weight loss temperature/°C
TG、DTG	Peak 1	43.3~172.2	5.02	—	100.7
	Peak 2	172.2~406.8	44.89	—	304.3
DTA	Peak 1	37.6–209.3	—	263.70	117.0
	Peak 2	321.3–410.1	—	41.59	357.7

The DTG of Shichian moss tea exhibited two peaks, with inflection points at 101.7°C and 337.1°C. The DTA of Shichian moss tea displayed a large exothermic peak with a peak value of 117.0°C, a temperature range of 37.6°C–209.3°C, and a peak area of 263.70 J/g; it also exhibited a heat‐absorbing peak with a peak value of 357.70°C, a temperature range of 321.3°C–410.1°C, and a peak area of 41.59 J/g.

#### 3.1.5 Pu'an Black Tea

The results of the TG analysis of the Pu‐an black tea are shown in Figure 2e, and the TG data are listed in Table 1e. The decomposition of the Pu'an black tea sample started at 39.7°C, and after the first stage of decomposition, the rate of loss was 7.06%, as shown in Figure 2e. When the temperature reached 185.2°C, it entered the second stage of decomposition, and the rate of loss was 37.97% when the temperature reached 408.4°C. The sample continued to decompose, and the final residual mass was 44.35%.

**FIGURE 2e:**
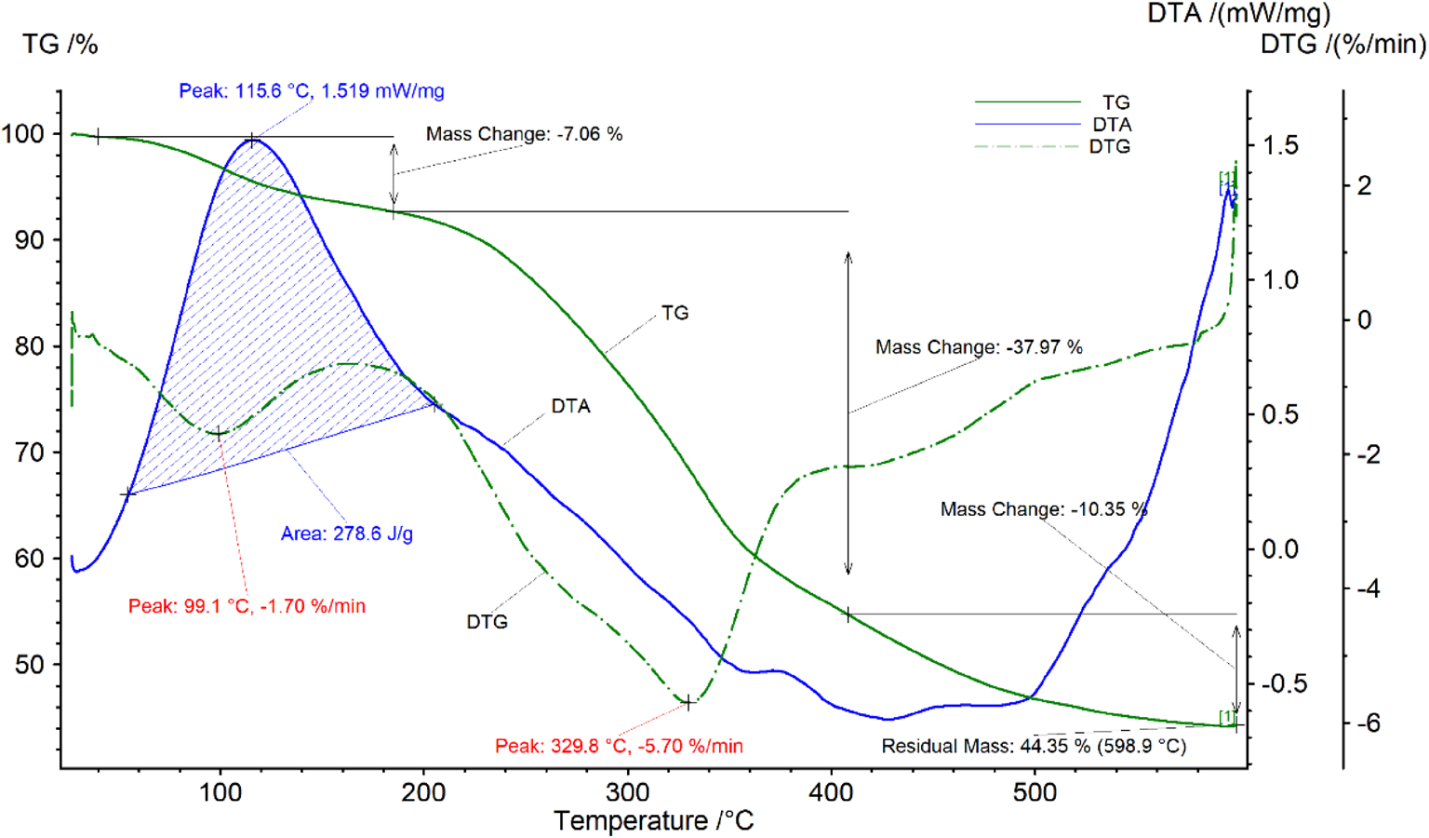
Thermogravimetric (TG) curve, thermogravimetric (DTG) curve and differential thermal analysis (DTA) curve of the Pu'an black tea.

**TABLE 1e:** Thermogravimetric analysis data of the Pu'an black tea.

Curve	Project	Temperature range/°C	Percentage weight loss/%	Peak area/(J/g)	The fastest weight loss temperature/°C
TG, DTG	Peak 1	39.7~185.2	7.06	—	99.1
	Peak 2	185.2~408.4	37.97	—	329.8
DTA	Peak 1	54.9~205.1	—	278.6	115.6

The DTG curve of Pu'an black tea showed two peaks, with inflection points at 99.1°C and 329.8°C. The DTA curve of Pu'an black tea had a large exothermic peak with a value of 115.6°C, a temperature range of 54.9°C–205.1°C, and a peak area of 278.6 J/g.

A combustion heat multi‐indicator evaluation system was developed using combustibility data from five types of tea: Qingyu tiny kuding tea, Guiding yunwu tribute tea, Suiyang mountain silver flower tea, Shiqian moss tea, and Pu'an black tea. This system employs a thermal weight analyzer to investigate the combustion characteristics of tea leaves at various heating rates to determine their combustion stability (Zhou, Jiang, Shi, and Jiang 2022). On the basis of gray pattern recognition methods (Ramachandran and Kathavarayan Ramu 2021; Rubin‐Falcone et al. 2018) and EXCEL analysis, the *F* values for these teas were recorded as follows: 0.8052 for Qingyu tiny kuding tea, 0.8095 for Guiding yunwu tribute tea, 0.8102 for Suiyang mountain silver flower tea, 0.8073 for Shiqian moss tea, and 0.8023 for Pu'an black tea. Accordingly, the teas were ranked by their combustion stability, with Suiyang mountain silver flower tea demonstrating the greatest stability, followed by Guiding yunwu tribute tea, Shiqian moss tea, Qingyu tiny kuding tea, and Pu'an black tea. TGA revealed distinct thermal decomposition onset (39.7°C–60.3°C) and final residual masses (18.39%–44.35%), with prominent exothermic peaks near 110°C–117°C (peak areas 201.90–282.00 J/g).

### 3.2 Determination of the Combustion Heat, Trace Element, Amino Acid, Fat, Ash, and Crude Fiber Contents

The results of the determination of the combustion heat, trace element, amino acid, fat, ash, and crude fiber contents of the five types of tea are presented in Table 2a. As was detected only in Suiyang mountain silver flower tea, whose concentration was 0.0369 μg/g (Table 2a). Hg and Sc were not detected in any of the five types of tea.

**TABLE 2a:** Determination results of the combustion heat, trace element, amino acid, fat, ash, and crude fiber contents of the five kinds of tea (*n* = 3, CV% < 2%).

Variables	Samples					
	Qingyu tiny kuding tea	Guiding yunwu tribute tea	Suiyang mountain silver flower tea	Shiqian moss tea	Pu'an black tea	*p*
*Q*_Vaverage_/(J/g)	6886.277 ± 194.248	2519.081 ± 117.817	4384.624 ± 182.984	6622.089 ± 272.035	3652.928 ± 104.167	0.196
Crude fiber/%	12.1954 ± 0.5889	12.3671 ± 0.5941	11.9916 ± 0.5676	11.1557 ± 0.3942	11.6968 ± 0.5333	0.000
Fat content/%	0.7433 ± 0.0338	0.275 ± 0.0081	0.2865 ± 0.0106	0.8532 ± 0.0298	0.4961 ± 0.0217	0.000
Ash/%	3.8309 ± 0.0238	6.0961 ± 0.0981	6.6598 ± 0.1181	5.7359 ± 0.059	5.5851 ± 0.0818	0.000
Al	98.8209 ± 0.0009	272.0509 ± 0.0006	56.4623 ± 0.0001	407.1322 ± 0.0070	582.3193 ± 0.0000	0.000
As	—	—	0.0369 ± 0.00001	—	—	
Ba	18.3861 ± 0.0002	7.1318 ± 0.0000	52.2895 ± 0.0008	20.3566 ± 0.0001	21.1883 ± 0.0003	0.000
Co	0.1105 ± 0.0002	0.0386 ± 0.0003	1.2186 ± 0.0002	0.2972 ± 0.0003	0.3221 ± 0.0001	0.000
Cr	2.2108 ± 0.0001	2.7371 ± 0.0001	3.0650 ± 0.0001	2.6746 ± 0.0001	0.8590 ± 0.0001	0.000
Cu	31.0243 ± 0.0003	25.6746 ± 0.0002	36.8907 ± 0.0004	35.9584 ± 0.0002	34.6457 ± 0.0003	0.000
Fe	396.0943 ± 0.0050	752.8913 ± 0.0050	327.2895 ± 0.0054	306.8351 ± 0.0005	144.9893 ± 0.0009	0.000
Hg	—	—	—	—	—	
Li	0.3316 ± 0.0000	0.1157 ± 0.0000	0.2216 ± 0.0000	0.1857 ± 0.000	0.1074 ± 0.0000	0.000
Mg	1109.0641 ± 0.0310	1340.7864 ± 0.0160	1618.1684 ± 0.0120	1649.3314 ± 0.0510	1679.6707 ± 0.0110	0.000
Mn	86.2196 ± 0.0011	96.4534 ± 0.0007	627.0310 ± 0.0110	699.8514 ± 0.0100	599.4989 ± 0.0100	0.000
Na	277.4134 ± 0.0009	325.5204 ± 0.0119	330.8715 ± 0.0067	296.1738 ± 0.0116	276.5211 ± 0.0343	0.000
Ni	2.1002 ± 0.0005	10.4472 ± 0.0001	6.4993 ± 0.0002	16.1590 ± 0.0001	8.5183 ± 0.0001	0.000
Pb	2.5055 ± 0.0008	1.8119 ± 0.0010	1.8095 ± 0.0008	2.3774 ± 0.0003	0.8948 ± 0.0016	0.000
Sc	—	—	—	—	—	
Sr	16.1017 ± 0.0002	2.1974 ± 0.0000	30.5022 ± 0.0003	7.9495 ± 0.0001	9.1625 ± 0.0001	0.000
Zn	164.1120 ± 0.0020	193.6777 ± 0.0027	848.2275 ± 0.0040	267.7192 ± 0.0038	122.2978 ± 0.0006	0.000
CySO_3_H	61.738 ± 0.4119	135.6587 ± 0.4869	203.7743 ± 1.8551	266.952 ± 0.2466	196.4393 ± 0.397	0.000
Asp	95.6023 ± 0.4771	1521.3037 ± 0.4191	3942.9303 ± 1.1408	4629.7443 ± 0.4531	650.1143 ± 0.4677	0.213
MetSON	—	—	—	—	—	
Thr	13.395 ± 0.1559	1589.8347 ± 5.0932	745.5683 ± 1.9586	857.7577 ± 0.8285	318.9537 ± 0.3629	0.000
Ser	51.115 ± 0.8667	1061.243 ± 3.1354	10,091.0533 ± 0.3795	13,527.2427 ± 1.4068	5767.878 ± 0.099	0.371
Glu	169.3513 ± 0.91	2162.7557 ± 0.7045	3812.4867 ± 1.8791	4227.7983 ± 0.4677	1132.4773 ± 0.4316	0.195
Gly	1.2263 ± 0.0095	24.612 ± 0.2778	26.9353 ± 0.4206	30.3793 ± 0.2587	3.2403 ± 0.0335	0.000
Ala	33.9633 ± 0.2177	551.8977 ± 1.8791	471.4107 ± 0.4783	412.927 ± 1.2966	513.02 ± 0.4797	0.000
(Cys)_2_	94.1117 ± 0.3625	5.656 ± 0.0195	55.3887 ± 0.3706	55.2343 ± 0.4486	93.9893 ± 0.9878	0.000
Val	430.1783 ± 0.4004	172.2103 ± 0.3795	1044.3697 ± 0.5413	814.942 ± 1.6214	653.8843 ± 1.4041	0.000
Met	266.0017 ± 0.4899	213.592 ± 0.4337	637.5553 ± 1.7773	647.7437 ± 0.3841	1143.7823 ± 0.1495	0.000
Ile	3.3293 ± 0.0335	167.4173 ± 1.5176	621.769 ± 0.4337	610.0623 ± 0.4153	290.9033 ± 0.1256	0.000
Leu	30.3597 ± 0.1436	239.6477 ± 0.5413	554.36 ± 2.0121	637.052 ± 0.1745	353.3603 ± 0.3528	0.000
Tyr	7.3453 ± 0.0404	204.7677 ± 1.6937	424.2467 ± 0.5413	684.583 ± 0.2063	704.5933 ± 0.6751	0.000
Phe	96.5593 ± 0.3706	321.6107 ± 0.3706	1139.458 ± 0.5589	1183.9047 ± 0.2991	668.1523 ± 0.4677	0.000
His	28.7077 ± 0.3136	78.9283 ± 0.4206	594.2147 ± 0.6031	412.4527 ± 0.1702	29.484 ± 0.144	0.000
Lys	37.9557 ± 0.6924	84.045 ± 1.2742	701.571 ± 0.5908	750.4003 ± 0.1695	21.6777 ± 0.1625	0.000
Arg	7492.729 ± 0.1623	224.3873 ± 1.2842	2020.5713 ± 1.1854	1272.8407 ± 0.0896	499.5183 ± 1.3004	0.420
Pro	—	1705.4873 ± 1.9734	494.0667 ± 0.4252	291.2137 ± 0.1049	165.7573 ± 1.9537	0.001
Thea	114.4494 ± 0.0015	18,603.8632 ± 0.0021	1171.8434 ± 0.0024	12,285.9776 ± 0.4315	5035.0768 ± 0.0106	0.186

MetSON was not detected in Shiqian moss tea, Suiyang mountain silver flower tea, Pu'an black tea, Guiding yunwu tribute tea, or Qingyu tiny kuding tea, and all five kinds of tea contained seven essential amino acids (Trp was not detected), namely, Thr, Val, Met, Ile, Leu, Phe, and Lys. The ratio of the essential amino acid content to the total amino acid content ranged from 9.78% to 26.20%, and the ratio of the essential amino acid content to the nonessential amino acid content ranged from 10.85% to 35.50%. The differences in the amino acid contents of the five teas may be related to the different climates, planting environments, and processing methods used in each tea‐producing area, and the amino acid compositions of the five teas were highly diverse.

### 3.3 Development of a Multi‐Indicator System for Evaluating the Nutritional Profile of Teas

#### 3.3.1 Entropy Factor Analysis

Entropy factor analysis (EFA) integrates information theory and factor analysis to reduce multivariate data and extract pertinent features (Eshima et al. 2018; Heng et al. 2023). The fundamental premise of EFA is the measurement of data complexity and uncertainty through entropy, which is then employed as a basis for conducting factor analysis to identify the most representative feature variables.

For the entropy factor analysis, the entropy coefficient of the sample is used to replace the sample correlation coefficient matrix in the factor analysis, which is used to participate in the computation and obtain the eigenvalues and eigenvectors, and the variance contribution rate and total variance contribution rate of the entropy factor analysis are computed (Shyu et al. 2011; Wang, Li, and Xie 2024).

After performing the entropy factor analysis, the cumulative contribution (Table 2b) of the first 4 entropy factors reached 100.00% > 85%, so the first 4 entropy factors were selected, which represented 100.00% of the 38 variables, including the combustion stability, *Q*_V_, ash, crude fiber, fat, Al, Ba Co, Cr, Cu, Fe, Li, Mg, Mn, Na, Ni, Pb, Sr., Zn, CySO_3_H, Asp, Thr, Ser, Glu, Gly, Ala, (Cys)_2_, Val, Met, Ile, Leu, and Tyr, Phe, His, Lys, Thea, Arg, and Pro, in five teas in Guizhou, China: Qingyu tiny kuding tea, Guiding yunwu tribute tea, Suiyang mountain silver flower tea, Shiqian moss tea, and Pu'an black tea.

**TABLE 2b:** Characteristic roots of the entropy correlation coefficients for the 38 variables in the five teas.

Entropy factors	Characteristic root	Contribution rate/%	Cumulative contribution rate/%
1	19.240	50.632	50.632
2	11.732	30.874	81.506
3	4.554	11.985	93.491
4	2.473	6.509	100.000

The first entropy factor loading matrix (Table 2c) contains information on Li, Mg, Mn, Pb, CySO_3_H, Ser, Ile, Leu, Tyr, and Phe. The content of Li was between 0.10 and 0.33 μg/g, with an average quality score of 0.1924 μg/g, and the content order was as follows: Qingyu tiny kuding tea > Suiyang mountain silver flower tea > Shiqian moss tea > Guiding yunwu tribute tea > Pu'an black tea.

**TABLE 2c:** The entropy factor loading matrix for the 38 variables in the five teas.

Variables	Entropy factor loading matrix 1	Entropy factor loading matrix 2	Entropy factor loading matrix 3	Entropy factor loading matrix 4
Combustion stability	−0.322	−0.760	−0.541	0.161
*Q*_V_	−0.473	0.786	0.188	−0.351
Crude fiber	−0.017	0.145	0.639	−0.755
Ash	0.590	0.742	0.035	0.317
Fat	−0.168	0.497	−0.388	−0.758
Al	−0.386	−0.848	0.262	0.252
Ba	−0.892	0.166	0.178	0.382
Co	−0.913	−0.181	0.218	0.292
Cr	−0.062	−0.758	−0.643	0.089
Cu	−0.990	0.114	0.019	−0.075
Fe	−0.326	−0.178	−0.928	0.029
Li	0.842	−0.436	−0.257	0.185
Mg	0.954	0.276	−0.059	0.103
Mn	0.957	−0.239	0.144	0.083
Na	−0.646	−0.746	−0.139	0.084
Ni	0.586	0.687	−0.042	−0.428
Pb	0.414	−0.491	−0.741	−0.196
Sr	−0.672	0.503	0.116	0.532
Zn	−0.042	−0.923	−0.093	0.372
CySO_3_H	0.927	0.360	0.096	−0.050
Asp	0.531	0.548	0.646	0.017
Thr	0.160	0.966	0.205	−0.005
Ser	0.955	−0.007	0.295	0.028
Glu	0.563	0.635	0.528	0.036
Gly	0.196	0.753	0.629	0.002
Ala	0.579	0.749	−0.259	0.190
(Cys)_2_	−0.652	0.545	−0.525	−0.043
Val	−0.985	0.014	0.171	0.009
Met	−0.874	−0.428	−0.191	−0.126
Ile	0.886	0.285	0.346	0.119
Leu	0.890	0.371	0.261	0.050
Tyr	0.975	0.168	−0.123	−0.074
Phe	0.935	0.176	0.291	0.099
His	0.260	0.523	0.808	0.076
Lys	−0.094	−0.990	0.080	−0.065
Thea	−0.139	−0.979	0.147	−0.026
Arg	−0.253	0.936	0.210	0.128
Pro	−0.041	0.979	0.052	0.192

The content of Mn ranged from 86.21 to 699.86 μg/g, with an average quality score of 421.8109 μg/g, and the content sequence was as follows: Shiqian moss tea > Suiyang mountain silver flower tea > Pu'an black tea > Guiding yunwu tribute tea > Qingyu tiny kuding tea.

The Pb content ranged from 0.89 to 2.51 μg/g, with an average quality score of 1.8798 μg/g, and the content sequence was as follows: Qingyu tiny kuding tea > Shiqian moss tea > Guiding yunwu tribute tea > Suiyang mountain silver flower tea > Pu'an black tea.

The concentration of CySO_3_H was between 61.73 and 266.96 μg/g, with an average quality score of 172.9125 μg/g, and the content order was Shiqian moss tea > Suiyang mountain silver flower tea > Pu'an black tea > Guiding yunwu tribute tea > Qingyu tiny kuding tea.

The content of Ser ranged from 51.11 to 13,527.25 μg/g, with an average quality score of 6099.7064 μg/g, and the order of content was as follows: Shiqian moss tea > Suiyang mountain silver flower tea > Pu'an black tea > Guiding yunwu tribute tea > Qingyu tiny kuding tea.

The Ile content ranged from 3.32 to 621.77 μg/g, with an average of 338.6962 μg/g, and the content sequence was as follows: Suiyang mountain silver flower tea > Shiqian moss tea > Pu'an black tea > Guiding yunwu tribute tea > Qingyu tiny kuding tea.

The Leu content ranged from 30.35 to 637.06 μg/g, with an average of 362.9559 μg/g. The Leu content decreased in the following order: Shiqian moss tea > Suiyang mountain silver flower tea > Pu'an black tea > Guiding yunwu tribute tea > Qingyu tiny kuding tea.

The content of tyr was found to range from 7.34 to 704.60 μg/g, with an average of 405.1072 μg/g, and the content order was as follows: Pu'an black tea > Shiqian moss tea > Suiyang mountain silver flower tea > Guiding yunwu tribute tea > Qingyu tiny kuding tea.

The content of Phe ranged from 96.55 to 1183.91 μg/g, with an average quality score of 681.9370 μg/g, and the content sequence was Shiqian moss tea > Suiyang mountain silver flower tea > Pu'an black tea > Guiding yunwu tribute tea > Qingyu tiny kuding tea.

The second entropy factor loading matrix contained information on *Q*_V_, ash, fat, Cu, Ni, Thr, Glu, Gly, Ala, (Cys)_2_, Arg, and Pro. The combustion heats of Guiding yunwu tribute tea, Suiyang mountain silver flower tea, Shiqian moss tea, and Pu'an black tea were in the following order: Qingyu tiny kuding tea > Shiqian moss tea > Suiyang mountain silver flower tea > Pu'an black tea > Guiding yunwu tribute tea. The combustion heat of the five teas ranged from 2519.08 to 6886.28 J/g, among which the highest energy was 6886.277 J/g (Qingyu tiny kuding tea), and the lowest energy was 2519.081 J/g (Guiding yunwu tribute tea), 4384.624 J/g (Suiyang mountain silver flower tea), 6622.089 J/g (Shiqian moss tea), and 3652.928 J/g (Pu'an black tea).

The ash content ranged from 3.83% to 6.66%, with an average content of 5.58%. The ash content decreased in the following order: Suiyang mountain silver flower tea > Guiding yunwu tribute tea > Shiqian moss tea > Pu'an black tea > Qingyu tiny kuding tea.

The fat content (%) of the five kinds of tea showed a range from 0.27% to 0.86%, with an average content of 0.53%, and the order of their fat content was Shiqian moss tea > Qingyu tiny kuding tea > Pu'an black tea > Suiyang mountain silver flower tea > Guiding yunwu tribute tea.

The content of Cu ranged from 25.67 to 36.90 μg/g, with an average of 32.84 μg/g, and the content sequence was as follows: Suiyang mountain silver flower tea > Shiqian moss tea > Pu'an black tea > Qingyu tiny kuding tea > Guiding yunwu tribute tea.

The Ni content ranged from 2.10 to 16.16 μg/g, with an average quality score of 8.7448 μg/g, and the content order was Shiqian moss tea > Guiding yunwu tribute tea > Pu'an black tea > Suiyang mountain silver flower tea > Qingyu tiny kuding tea.

The Thr content showed a range from 13.39 to 1589.84 μg/g, with an average of 705.1019 μg/g, and the content order was as follows: Guiding yunwu tribute tea > Shiqian moss tea > Suiyang mountain silver flower tea > Pu'an black tea > Qingyu tiny kuding tea.

The Glu concentration ranged from 169.35 to 4227.80 μg/g, with an average quality score of 2300.9739 μg/g. The Glu content decreased in the following order: Shiqian moss tea > Suiyang mountain silver flower tea > Guiding yunwu tribute tea > Pu'an black tea > Qingyu tiny kuding tea.

The content of Gly showed a range from 1.22 to 30.38 μg/g, with an average quality score of 17.2786 μg/g, and the content order was Shiqian moss tea > Suiyang mountain silver flower tea > Guiding yunwu tribute tea > Pu'an black tea > Qingyu tiny kuding tea.

The Ala concentration ranged from 33.96 to 551.90 μg/g, with an average of 396.6437 μg/g, and the content order was as follows: Guiding yunwu tribute tea > Pu'an black tea > Suiyang mountain silver flower tea > Shiqian moss tea > Qingyu tiny kuding tea.

The content of (Cys)_2_ ranged from 5.65 to 94.12 μg/g, with an average of 60.8760 μg/g, and the order of content was as follows: Qingyu tiny kuding tea > Pu'an black tea > Suiyang mountain silver flower tea > Shiqian moss tea > Guiding yunwu tribute tea.

The content of Arg ranged from 224.38 to 7492.73 μg/g, with an average of 2302.0093 μg/g. The content decreased in the following order: Qingyu tiny kuding tea > Suiyang mountain silver flower tea > Shiqian moss tea > Pu'an black tea > Guiding yunwu tribute tea.

The content of Pro showed a range from 165.75 to 1705.49 μg/g, with an average of 531.3050 μg/g, and the content order was as follows: Guiding Yunwu tribute tea > Suiyang mountain silver flower tea > Shiqian moss tea > Pu'an black tea. Pro was not detected in the tiny kuding tea of Qingyu.

The third entropy factor loading matrix contained information on crude fiber, Al, Cr, Asp, Val, His, Lys, and Thea. The crude fiber content (%) ranged from 11.15% to 12.37%, with an average content of 11.88%. The order of crude fiber content was as follows: Guiding yunwu tribute tea > Qingyu tiny kuding tea > Suiyang mountain silver flower tea > Pu'an black tea > Shiqian moss tea.

The content of Al was between 98.82 and 582.32 μg/g, with an average of 283.3571 μg/g, and the content order was as follows: Pu'an black tea > Shiqian moss tea > Guiding yunwu tribute tea > Qingyu tiny kuding tea > Suiyang mountain silver flower tea.

The content of Asp ranged from 95.60 to 4629.75 μg/g, with an average of 2167.9390 μg/g, and the order of Asp content was Shiqian moss tea > Suiyang mountain silver flower tea > Guiding yunwu tribute tea > Pu'an black tea > Qingyu tiny kuding tea.

The Val content showed a range from 172.21 to 1044.37 μg/g, with an average quality score of 623.1169 μg/g, and the content order was as follows: Suiyang mountain silver flower tea > Shiqian moss tea > Pu'an black tea > Qingyu tiny kuding tea > Guiding yunwu tribute tea.

The His content ranged from 28.70 to 594.22 μg/g, with an average of 228.7575 μg/g, and the His content decreased in the following order: Suiyang mountain silver flower tea > Shiqian moss tea > Guiding yunwu tribute tea > Pu'an black tea > Qingyu tiny kuding tea.

The Lys concentration ranged from 21.67 to 750.41 μg/g, with an average of 319.1299 μg/g. The Lys content decreased in the following order: Shiqian moss tea > Suiyang mountain silver flower tea > Guiding yunwu tribute tea > Qingyu tiny kuding tea > Pu'an black tea.

The content of Thea ranged from 114.44 to 18,603.87 μg/g, with an average of 7442.2421 μg/g, and the content sequence was as follows: Guiding yunwu tribute tea > Shiqian moss tea > Pu'an black tea > Suiyang mountain silver flower tea > Qingyu tiny kuding tea.

The fourth entropy factor loading matrix contained information on the combustion stability, Ba Co, Fe, Na, Sr., Zn, and Met. The Ba concentration ranged from 7.13 to 52.29 μg/g, with an average quality score of 23.8705 μg/g, and the content sequence was as follows: Suiyang mountain silver flower tea > Pu'an black tea > Shiqian moss tea > Qingyu tiny kuding tea > Guiding yunwu tribute tea.

The Co concentration ranged from 0.03 to 1.21 μg/g, with an average quality score of 0.3974 μg/g, and the content sequence was as follows: Suiyang mountain silver flower tea > Pu'an black tea > Shiqian moss tea > Qingyu tiny kuding tea > Guiding yunwu tribute tea.

The Fe content ranged from 144.98 to 752.90 μg/g, with an average quality score of 385.6199 μg/g, and the order of Fe content was as follows: Guiding yunwu tribute tea > Qingyu tiny kuding tea > Suiyang mountain silver flower tea > Shiqian moss tea > Pu'an black tea.

The concentration of Na was between 276.52 and 330.88 μg/g, with an average value of 301.3000 μg/g, and the content order was as follows: Suiyang Mountain silver flower tea > Guiding yunwu tribute tea > Shiqian moss tea > Qingyu tiny kuding tea > Pu'an black tea.

The Sr content ranged from 2.20 to 30.51 μg/g, with an average of 13.1827 μg/g, and the order of the Sr content was as follows: Suiyang mountain silver flower tea > Qingyu tiny kuding tea > Pu'an black tea > Shiqian moss tea > Guiding yunwu tribute tea.

The Zn content ranged from 122.30 to 848.23 μg/g, with an average quality score of 319.2068 μg/g, and the content order was as follows: Suiyang mountain silver flower tea > Shiqian moss tea > Guiding yunwu tribute tea > Qingyu tiny kuding tea > Pu'an black tea.

The Met content ranged from 213.59 to 1143.79 μg/g, with an average of 581.7350 μg/g, and the content sequence was as follows: Pu'an black tea > Shiqian moss tea > Suiyang mountain silver flower tea > Qingyu tiny kuding tea > Guiding yunwu tribute tea.

This ranking was derived from the composite entropy factor scores, which were calculated using the weighted least‐squares approach, with weights being the eigenroots of the entropy factors (*F*_1_–*F*_4_) and composite entropy factor scores (Yin et al. 2023; Zhang et al. 2024; Zou et al. 2024) (*F*), with the weights being the eigenroots of the entropy factors (Seo et al. 2024; Wang, Peng, et al. 2024). The formula used was *F* = 0.5063 *F*_1_ + 0.3087 *F*_2_+ 0.1199 *F*_3_+ 0.0651 *F*_4_. The entropy factor scores and composite entropy factor scores (Table 2d) of the 38 variables, namely, combustion stability, *Q*_V_, ash, crude fiber, fat, Al, Ba Co, Cr, Cu, Fe, Li, Mg, Mn, Na, Ni, Pb, Sr., Zn, CySO_3_H, Asp, Thr, Ser, Glu, Gly, Ala, (Cys)_2_, Val, Met, Ile, Leu, Tyr, Phe, His, Lys, Thea, Arg, and Pro, revealed that the following order of the entropy factor scores of the five teas were as follows: Qingyu tiny kuding tea > Shiqian moss tea > Suiyang mountain silver flower tea > Pu'an black tea > Guiding yunwu tribute tea. In this study, the observed differences in amino acid composition, trace element accumulation, and thermal properties among the five tea samples are likely due to complex interactions between cultivar‐specific traits and environmental factors. The distinct genetic backgrounds of Qingyu tiny kuding tea, Guiding yunwu tribute tea, Suiyang mountain silver flower tea, Shiqian moss tea, and Pu'an black tea may intrinsically regulate nitrogen metabolism, amino acid biosynthesis, and protein turnover, thereby determining their characteristic amino acid profiles (Jiang et al. 2021). These cultivar‐related effects are further influenced by geographical origin and local environmental conditions, such as altitude, temperature, rainfall, and light intensity, which can affect leaf growth rates, photosynthetic activity, and the partitioning of nitrogen between free amino acids and structural proteins (Deng et al. 2024; Kamal et al. 2025).

**TABLE 2d:** Entropy factor scores and composite entropy factor scores for the 38 variables in the five kinds of tea.

Sample	*F*_1_	*F*_2_	*F*_3_	*F*_4_	*F*	Ranking
Qingyu tiny kuding tea	1.2273	1.2614	−0.2427	0.2088	0.9953	1
Guiding yunwu tribute tea	−1.5447	0.8347	−0.3072	−0.1513	−0.5711	5
Suiyang mountain silver flower tea	−0.1154	−0.5521	1.0142	1.3614	−0.0186	3
Shiqian moss tea	0.2340	−0.4438	0.9320	−1.4421	−0.0007	2
Pu'an black tea	0.1988	−1.1002	−1.3963	0.0232	−0.4049	4

Similarly, the observed variation in trace element content among the teas is plausibly associated with differences in the soil geochemistry and root uptake capacity of each cultivar. Soils with contrasting pH, organic matter content, and mineral composition across the producing regions of Guizhou can alter the bioavailability of macro‐ and microelements, leading to distinct elemental fingerprints in the harvested leaves. Agricultural practices, including fertilization regimes and potential exposure to anthropogenic inputs, may further accentuate or attenuate these patterns (Hoang et al. 2026; Xu et al. 2024). Collectively, these cultivar‐specific and environmental mechanisms provide a coherent explanation for the multiparameter differences identified by our chemometric evaluation (Kenzo et al. 2024).

The results of the multi‐indicator evaluation of 38 variables indicated that Qingyu tiny kuding tea exhibited the highest quality, followed by Shiqian moss tea. Suiyang mountain silver flower tea ranked third in quality, whereas Pu'an black tea and Guiding yunwu tribute tea ranked fourth and fifth, respectively. These findings suggest that the distinct characteristics and composition of each tea variety significantly contribute to its overall quality and potential health benefits. Further research should investigate the specific compounds and processing methods that contribute to the superior quality of Qingyu tiny kuding tea and Shiqian moss tea.

#### 3.3.2 Entropy Factor Cluster Analysis (EFCA)

Entropy factor cluster analysis (EFCA) is a statistical approach that groups variables according to their similarities or relationships. It is a combination of cluster analysis and entropy factor analysis, whereby the variables are initially reduced to a smaller number of factors via entropy factor analysis and then clustered on the basis of their similarities via cluster analysis (Gupta and Dixit 2023; Han et al. 2024; Sun 2024).

On the basis of the 38 variables, including combustion stability, *Q*_V_, ash, crude fiber, fat, Al, Ba Co, Cr, Cu, Fe, Li, Mg, Mn, Na, Ni, Pb, Sr., Zn, CySO_3_H, Asp, Thr, Ser, Glu, Gly, Ala, (Cys)_2_, Val, Met, Ile, Leu, Tyr, Phe, His, Lys, Thea, Arg, and Pro, in five teas in Guizhou, China: Qingyu tiny kuding tea, Guiding yunwu tribute tea, Suiyang mountain silver flower tea, Shiqian moss tea, and Pu'an black tea. The five tea samples are shown in Figure 3a, with EFCA indicating that these samples can be categorized into three distinct groups. Specifically, Suiyang mountain silver flower tea, Shiqian moss tea, and Pu'an black tea were classified into one group, Guiding yunwu tribute tea formed another group, and Qingyu tiny kuding tea constituted the final group, indicating substantial variations in the chemical compositions and processing methods among these groups. The first group, consisting of three varieties, may have similar fermentation processes and regional cultivation conditions. In contrast, Guiding yunwu tribute tea, which formed a separate cluster, likely displayed distinct oxidative profiles and varietal characteristics. Qingyu tiny kuding tea, which constitutes its own distinct group, probably differs significantly in terms of bioactive compounds and processing techniques, reflecting its classification as a traditional tea (Zhang et al. 2025). OPLS–DA (Jin et al. 2024; Jin et al. 2023) was conducted to examine the relationships between the 38 variables and the five types of tea. The score matrix is shown in Figure 3b, and an analytical discriminant model was developed to differentiate the five teas on the basis of the 38 variables. The fitting parameters of the model were *R* ^2^ *X* = 0.887, *R* ^2^ *Y* = 0.998, and *Q* ^2^ = 0.997. To determine whether the model was overfit, the OPLS–DA model was validated by replacing parameter 200, and the results are shown in Figure 3c. Using leave‐one‐out cross‐validation, which yielded an overfitting rate of 0%, the *Q*_2_ value of −0.75 (< 0.05) indicates that the model is not overfit and effectively mitigates concerns regarding overfitting and insufficient validation (Lin et al. 2024). In conjunction with Figure 3d, when the selection criterion of VIP > 1 was used, the VIP values for Pro, Arg, Ala, Cu, Thr, Mn, Mg, Fe, (Cys)_2_, CySO_3_H, Tyr, Ash, Li, Leu, Val, Phe, Ser, Ile, *Q*_V_, Met, and Crude fiber were 1.28384, 1.25411, 1.25277, 1.23187, 1.2284, 1.22663, 1.22428, 1.20348, 1.17393, 1.17122, 1.15957, 1.15551, 1.14674, 1.12581, 1.12193, 1.12156, 1.10036, 1.07676, 1.06899, 1.02211, and 1.00684, respectively, all of which exceeded 1.00000. OPLS–DA revealed significant differences among the five tea types, with 21 components recognized as key differentiators of the tea types.

**FIGURE 3a:**
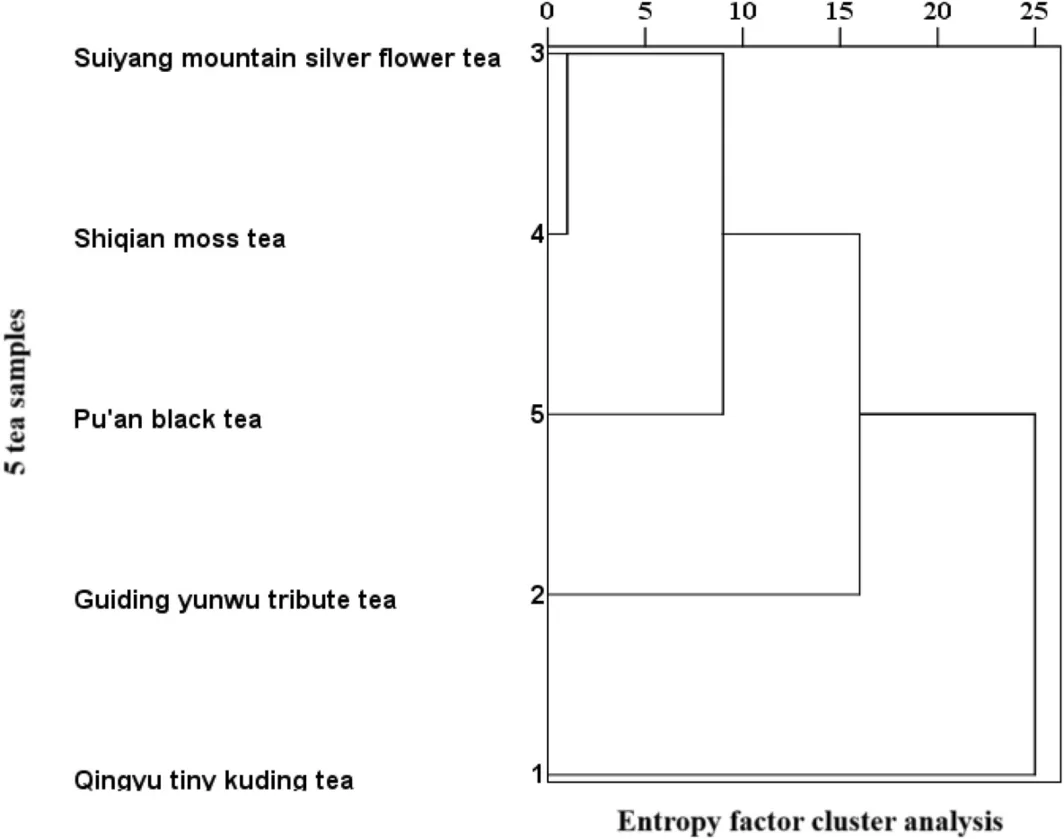
Entropy factor cluster analysis (EFCA) of five tea samples.

**FIGURE 3b:**
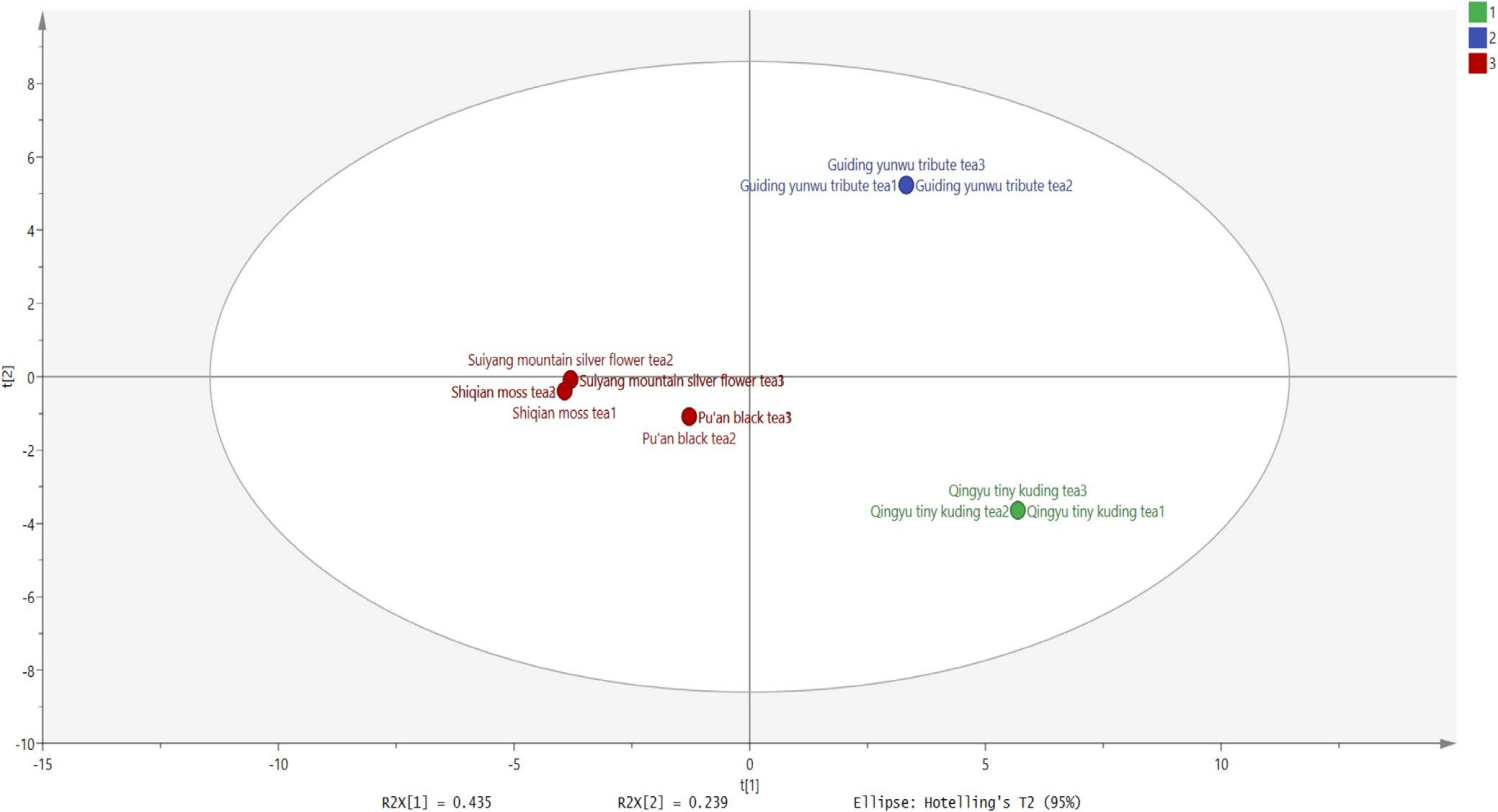
OPLS−DA scores of 38 variables of the five teas.

**FIGURE 3c:**
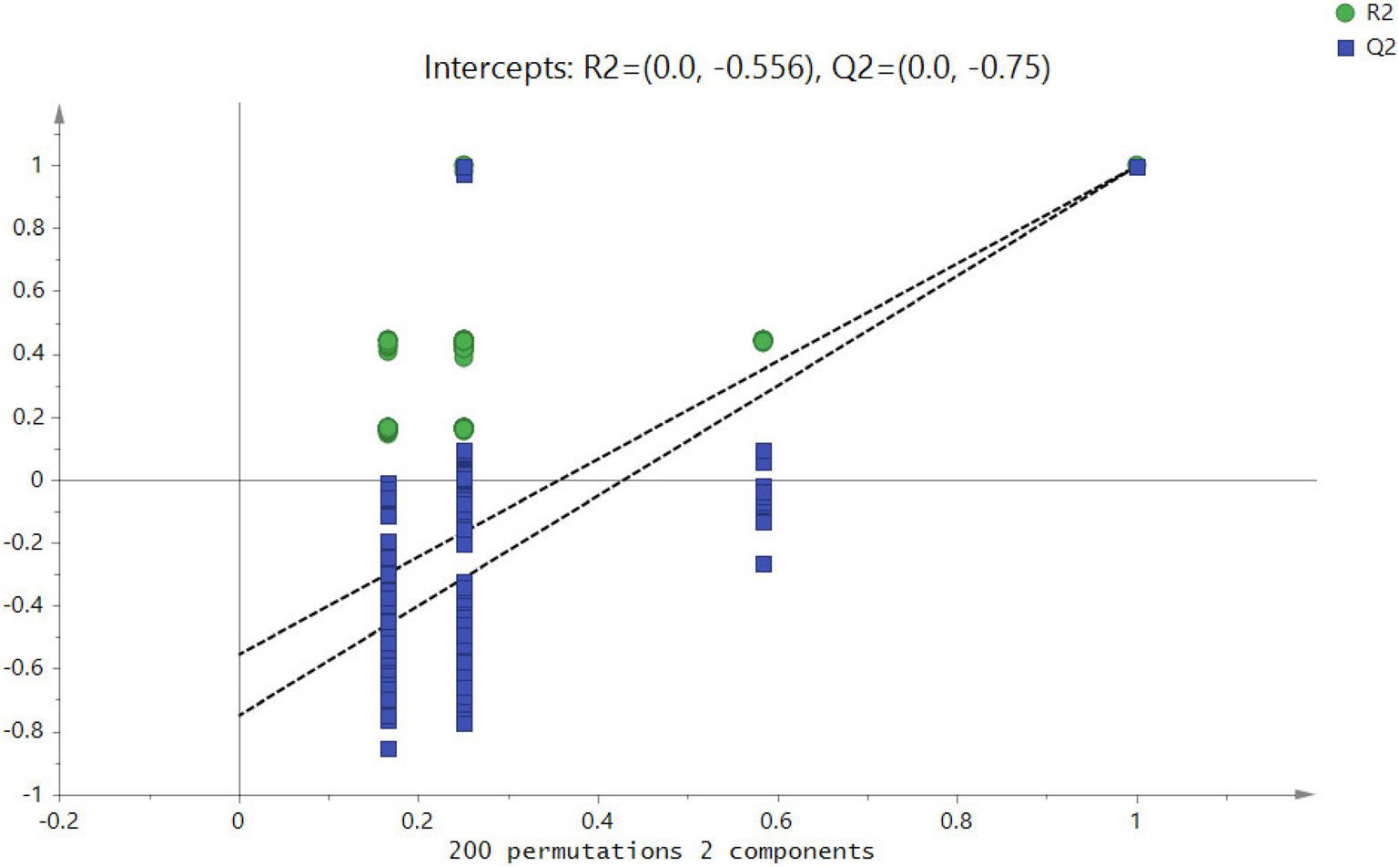
OPLS–DA replacement detection results.

**FIGURE 3d:**
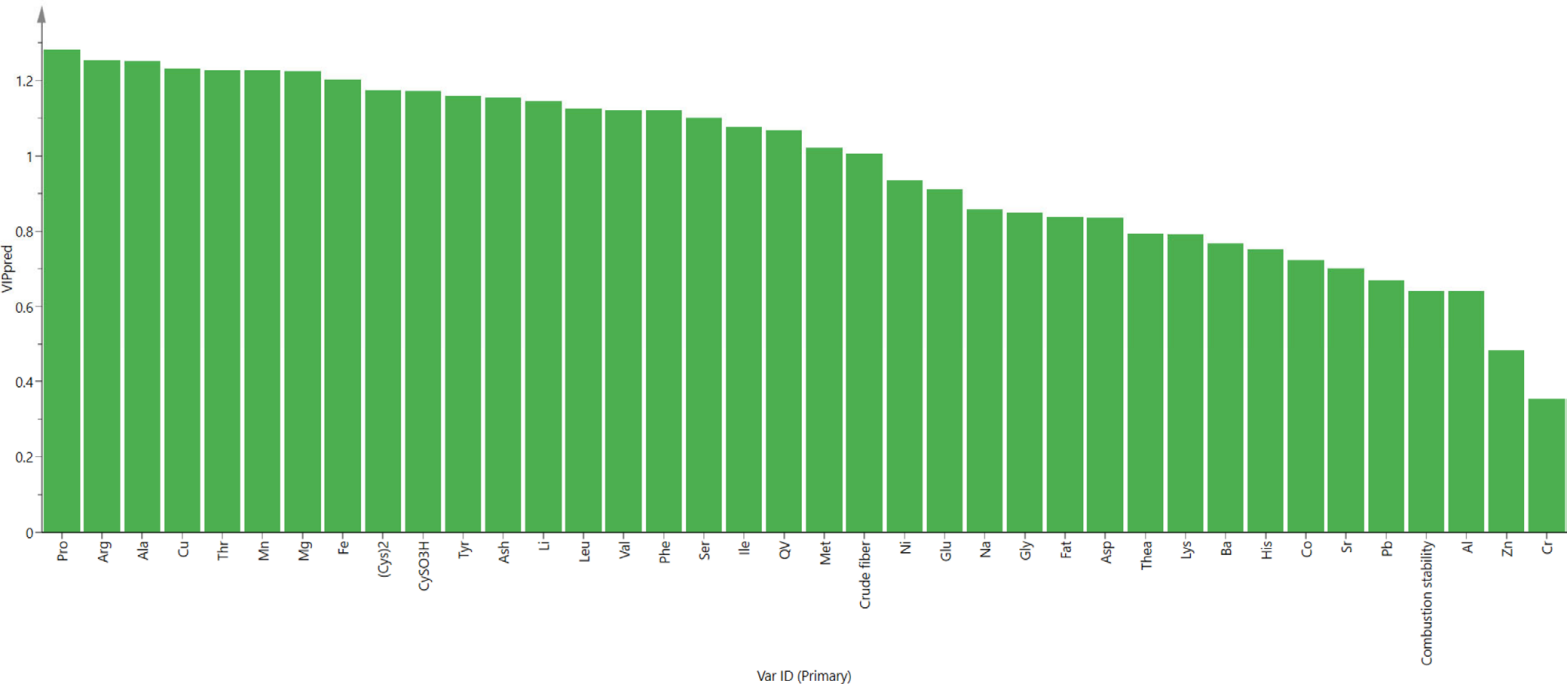
OPLS−DA VIPs of 38 variables of the five teas.

In this study, the 21 variables identified by the OPLS–DA method served as essential chemometric markers for effectively distinguishing between the five tea samples. The coordinated variation in these variables reflects the combined influence of cultivar traits and environmental conditions on amino acid composition (Graziano et al. 2019), trace element accumulation, and thermal behavior, facilitating a more nuanced classification than single‐parameter comparisons do. These components, including amino acids and structural elements, are crucial in defining the unique characteristics of each tea variety. Further investigations into the specific contributions of these markers could provide valuable insights into tea‐processing techniques and regional cultivation practices. This comprehensive analysis not only enhances our understanding of tea composition but also offers potential applications in the quality control and authentication of tea products.

An entropy factor cluster analysis (EFCA) was conducted on 38 variables based on various indicators. As shown in Figure 3e, the 38 variables were classified into three categories. The variables Ile, Phe, CySO_3_H, Leu, Mg, Tyr, ash, Ala, Mn, Ni, Ser, and Li were clustered into one category. The first group of variables appears to be primarily composed of essential amino acids and minerals, indicating a potential correlation between these components within the analyzed samples. Cu, Val, Ba Co, Sr., Asp, Glu, Gly, His, Arg, Pro, Thr, *Q*_V_, fat, (Cys)_2_, and crude fiber were clustered into one category. The second cluster comprises a combination of amino acids, minerals, and macronutrients, which may suggest a more comprehensive nutritional profile or functional characteristics. Similarly, Met, Lys, Thea, Al, Zn, combustion stability, Fe, Cr, Pb, and Na were also grouped together in a single category. The third group included sulfur‐containing amino acids, metals, and stability factors, potentially indicating interactions among these components that influence the overall quality or characteristics of the samples (Meng et al. 2024).

**FIGURE 3e:**
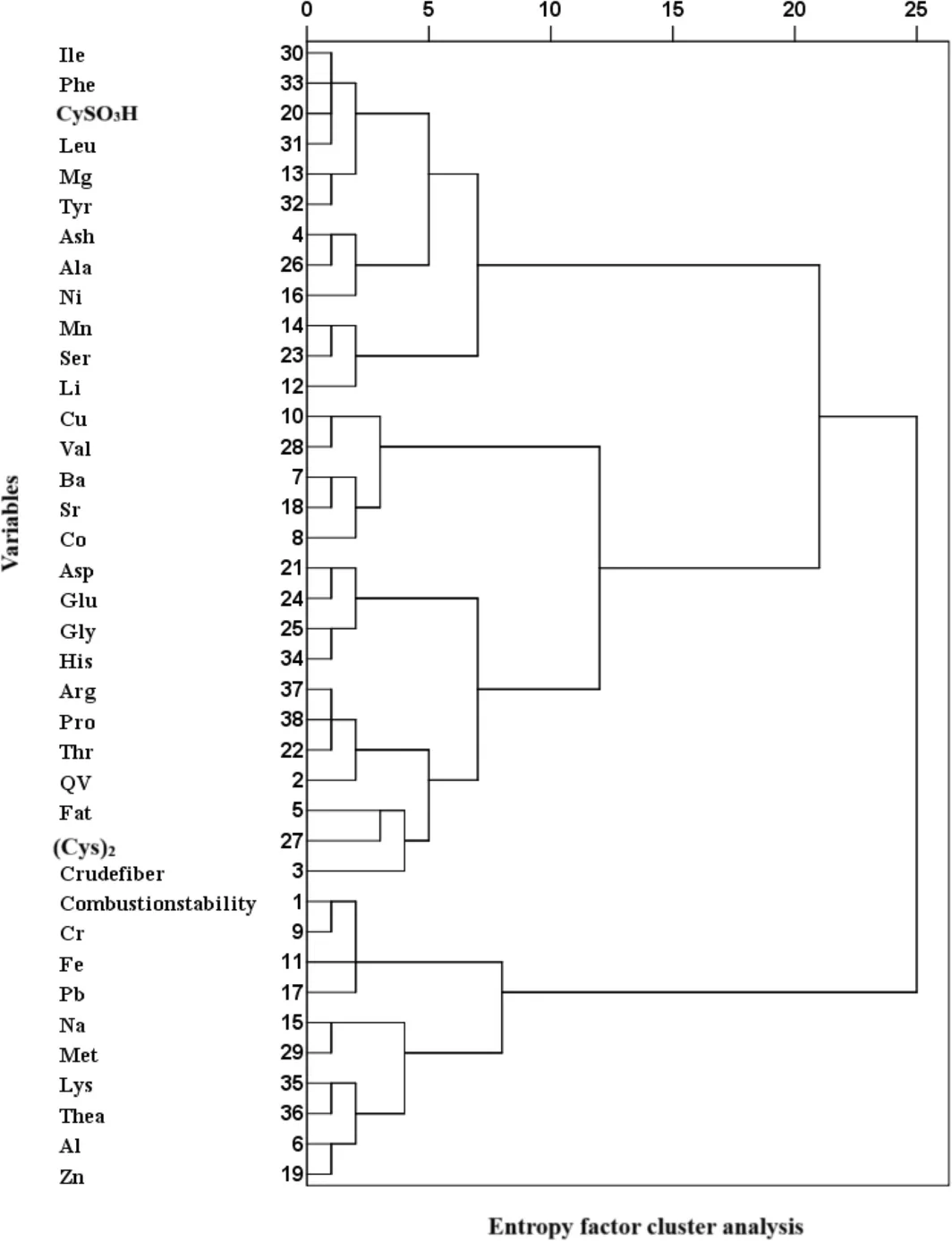
Entropy factor cluster analysis (EFCA) for 38 variables of the five teas.

This study employed thermogravimetric properties, amino acid profiles, and trace element characteristics as a comprehensive multiparameter framework for the discrimination and characterization of five tea samples. Amino acid indicators primarily capture sensory and nutritional attributes. Trace element patterns reflect mineral and environmental signatures linked to soil geochemistry, fertilization, and local growing conditions, thereby facilitating geographical and cultivar‐related differentiation (Johnson et al. 2010). Thermogravimetric parameters include thermal stability and compositional features, with variations in decomposition temperatures, mass loss stages, and residual mass indicating differences in organic matrix composition, moisture binding, and inorganic content. The integration of these indicator groups in chemometric analysis allows for the reliable separation of teas that are similar in one dimension by another, thereby enhancing the robustness of tea quality discrimination and providing a more mechanistic understanding of the observed differences among samples (Zhou, Jiang, Shi, and Jiang 2022).

## 4 Conclusion

This study addresses the limitations of traditional sensory or single‐indicator tea evaluation by developing an objective, multidimensional quality/nutrition assessment system for five Guizhou (China) teas—Qingyu tiny kuding tea, Guiding yunwu tribute tea, Suiyang mountain silver flower tea, Shiqian moss tea, and Pu'an black tea—integrating thermogravimetric/combustion behavior with amino acid and trace element profiles plus proximate indices (ash, crude fiber, fat, and combustion heat). Market samples were authenticated, dried (85°C, 12 h), ground (40‐mesh), and measured in triplicate (*n* = 3; RSD/CV < 2%) using an STA 2500 TGA (TG/DTG/DTA), an A300 amino acid analyzer (20 amino acids quantified from HCl extracts at 570/440 nm), and ICP–OES for elements, with the combustion heat and other proximate indices determined per established protocols. TGA revealed distinct thermal behaviors across teas, including decomposition onset spanning 39.7°C–60.3°C, two DTG peaks per sample (inflection points at ~89°C–108°C and ~316°C–337°C), prominent exothermic events near 110°C–117°C (peak areas of 201.90–282.00 J/g), and final residual masses ranging from 18.39% (Guiding yunwu tribute tea) to 44.35% (Pu'an black tea), indicating compositional differences in the organic matrix, moisture binding, and inorganic residue. Gray pattern recognition quantified the combustion stability (*F* = 0.8102–0.8023), with Suiyang mountain silver flower tea being the best, followed by Guiding yunwu tribute tea, Shiqian moss tea, Qingyu tiny kuding tea, and Pu'an black tea. Elemental screening revealed that As was detected only in Suiyang mountain silver flower tea (0.0369 μg/g), whereas Hg and Sc were not detected in any sample; amino acid profiling revealed that MetSON was absent in all five teas, and all samples contained seven essential amino acids (Thr, Val, Met, Ile, Leu, Phe, Lys; and Trp were not detected), with essential/total amino acid ratios of 9.78%–26.20% and essential/nonessential ratios of 10.85%–35.50%, underscoring strong varietal/environmental influences. For comprehensive quality ranking, entropy factor analysis reduced 38 variables (combustion stability; combustion heat; ash, fat, and crude fiber; 17 elements, including Pb; and 20 amino acids, including theanine) to four entropy factors explaining 100% of the variance, which were combined as *F* = 0.5063*F*_1_ + 0.3087*F*_2_ + 0.1199*F*_3_ + 0.0651*F*_4_; this multi‐indicator system yielded an overall quality order of Qingyu tiny kuding tea (highest) > Shiqian moss tea > Suiyang mountain silver flower tea > Pu'an black tea > Guiding yunwu tribute tea (reported composite scores *F*≈−0.5711 to 0.9953). Entropy factor cluster analysis further separated the five teas into three sample clusters (Suiyang/Shiqian/Pu'an together; Guiding alone; Qingyu alone) and grouped the 38 variables into three variable clusters reflecting coordinated patterns among amino acids, minerals/macronutrients, and stability/metal‐related indicators. Supervised OPLS–DA provided strong discrimination among tea types (*R* ^2^ *X* = 0.887, *R* ^2^ *Y* = 0.998, and *Q* ^2^ = 0.997), with permutation testing indicating no overfitting, and identified 21 key differentiators (VIP > 1): Pro, Arg, Ala, Cu, Thr, Mn, Mg, Fe, (Cys)_2_, CySO_3_H, Tyr, ash, Li, Leu, Val, Phe, Ser, Ile, combustion heat (*Q*_V_), Met, and crude fiber. Collectively, the results of this work demonstrate that integrating thermogravimetric parameters, amino acid composition, and elemental fingerprints within a chemometric framework improves the sensitivity and interpretability of tea quality classification, providing a practical scientific basis for Guizhou tea resource development, authentication/classification, and multi‐index quality control and market positioning.

This study has several limitations that should be acknowledged. First, the number of tea samples and cultivars examined was relatively small, and all the samples were sourced from the Guizhou region, which may limit the generalizability of the findings to other tea‐producing areas. Second, although the proposed chemometric framework integrating thermogravimetric behavior, amino acid composition, and trace element profiles shows promise for quality evaluation and discrimination, its robustness requires further validation using a larger and more diverse sample set that includes additional regions, cultivars, and processing types. Future research will involve expanding the geographic and varietal coverage, systematically testing the framework in targeted authentication and origin‐tracing scenarios, and comparing its performance with existing quality‐assessment methods. Additionally, additional samples will be collected to test additional parameters, including catechins, caffeine, polyphenols, and aroma compounds, and for sensory evaluation.

In this study, we constructed comprehensive evaluation systems for five types of tea based on nutritional indicators, including multiple indicators of the heat of combustion, combustibility (tea combustion stability), fat content, amino acid content, ash content, crude fiber content, and trace element content, using gray pattern recognition (GPR), entropy factor analysis (EFA), and entropy factor cluster analysis (EFCA). This study has significant theoretical and practical implications for the determination and comprehensive evaluation of multiple tea quality indicators. This study provides a scientific basis and research significance for nutritional, health, and classification studies of tea in Guizhou, China.

## Author Contributions

**Libing Zhou:** investigation, methodology, writing – review and editing, writing – original draft, funding acquisition. **Chunli Huang:** investigation, writing – original draft, writing – review and editing.

## Funding

This research was supported by the High‐Level Talents Project of Guangxi Science & Technology Normal University (GXKS2020GKY006) and Guangxi Science & Technology Normal University Key Laboratory of Speciality Food Evaluation and Application (GXKSKYPT2024011).

## Ethics Statement

Ethical approval was not needed. The five teas were purchased from Guiyang Yunyan district farmers' markets in Guizhou, China. This article does not contain any studies involving human participants or animals performed by the authors.

## Conflicts of Interest

The authors declare no conflicts of interest.

## Data Availability

All the authors have read and approved the final version of the manuscript. Libing Zhou, the corresponding author, has full access to all the data in this study and takes complete responsibility for the integrity of the data and the accuracy of the data analysis.
